# A New Method for Quantitative Evaluation Concentration Polarization Under Different Conditions for the Forward Osmosis Process

**DOI:** 10.3390/membranes15080223

**Published:** 2025-07-25

**Authors:** Ping Xiao, Liang Liu

**Affiliations:** Green Intelligence Environmental School, Yangtze Normal University, No. 16 Juxian Road, Fuling District, Chongqing 408100, China; xiaoping19831020@126.com

**Keywords:** forward osmosis, water transmission coefficient, osmotic pressure difference, water flux

## Abstract

Concentration polarization (CP) is one of the inherent problems that lowers the operating performance of forward osmosis (FO) membranes. Therefore, a quantitative evaluation of CP is vital to understand its impact on the FO process. This study systematically investigated the influences of different CPs on the osmotic pressure drop across the membrane under different conditions by using the water transmission coefficient, η_WT_, defined as the ratio of the measured water flux to the theoretical water flux. The results showed that η_WT_ decreased with an increase in the concentration gradient between the draw solution (DS) and the feed solution (FS) under different conditions. The proportions of osmotic pressure drop caused by dilutive internal concentration polarization (ICP) increased, while those caused by concentrative external concentration polarization (ECP) decreased, in different types of DSs in FO mode. Both ECP and ICP were found to be capable of reducing osmotic pressure. However, the internal CP had the dominant influence. To better understand the adverse effects of CP, using an organic FS provided greater insight than using deionized (DI) water as the FS. As the FS concentration increased, the water flux reduced, and the adverse effects of CP worsened. CaCl_2_ led to a greater reduction in water transfer efficiency than NaCl when used as the DS. In comparison to FO mode, pressure-retarded osmosis (PRO) mode led to greater pure water flux and flux decline. In FO mode, both the proportion of dilutive ICP and the η_WT_ decreased, while the proportion of concentrative ECP increased over time. However, in PRO mode, the proportions of dilutive ECP and concentrative ICP increased, and η_WT_ gradually decreased. Dilutive ICP had a significant negative effect on osmotic pressure in the former, while dilutive ECP was dominant in the latter.

## 1. Introduction

Forward osmosis (FO), as an alternative to pressure-driven membrane processes, has been widely used in the field of desalination and wastewater treatment. The driving force for the FO process, which happens spontaneously, is provided by the osmotic pressure difference across the membrane. It has been found that the experimental water flux is much lower than the theoretical water flux. Previous studies have shown that the experimental water flux under different conditions was about 0.5–90% of the theoretical water flux [1]. This significant discrepancy is caused by the cessation of perfect semi-permeability, concentration polarization (CP), reverse solute flux, and membrane fouling [2,3,4,5,6]. CP in the FO membrane is the main cause of the significant reduction in the effective osmosis pressure differential, resulting in a rapid decline in water flux [7,8]. Most of the FO membranes, including a dense active layer and a porous support layer, are asymmetric. Therefore, CP is inevitable and generally appears on both sides of the membrane. It can be further classified as external concentration polarization (ECP), occurring at the surface of the membrane active layer, and internal concentration polarization (ICP), which is exclusive to FO in the membrane support layer.

Previous research has identified ICP as the primary cause of the reduction in water flux by more than 80%, while external CP (ECP) plays a minor role in this reduction [8]. The increased flow rate significantly reduces the negative influence of ECP, improves the effective osmotic pressure difference to a certain extent, and subsequently promoted the water flux of the membrane [9,10,11]. However, ICP is affected by structural parameters such as the thickness, porosity, and tortuosity of the membrane support layer [12,13]. Therefore, the most common method for mitigating the ICP consists of modifying the structure of the FO membrane or preparing a new symmetric FO membrane with two active layers [14,15,16,17,18,19]. Alihemati et al. designed a double-skinned thin-film composite (TFC) and thin-film nanocomposite (TFN) hollow fiber membrane for FO [14]. By adding the second active layer to the substrate, water flux, reverse salt flux, and ICP decreased. To compensate for the decrease in water flux in the double-skinned membrane, graphene oxide (GO) nanoparticles were added to the outer active layer to improve water flux without negating the reverse salt flux. The novel double-skinned TFN hollow fiber membrane exhibited high FO performance with low ICP and fouling. Zhao et al. successfully developed a novel double-skinned FO membrane [15], which significantly reduced ICP and exhibited higher flux when using DSs with high concentrations compared to the single-skinned FO membrane.

Solution diffusion and diffusive convective transport in porous media have been used to describe FO membrane performance and evaluate the effects of CP based on different assumptions [20,21]. In these models, the actual driving force can be calculated using the osmotic reflection coefficient, which is assumed to be 1. This means that no solute can enter the membrane, and the membrane is semi-permeable. This assumption cannot explain reverse salt penetration. Therefore, the unclear definition of the actual driving force is a major drawback of these models. Many studies do not assume the reflection coefficient is 1; instead, solute rejection is explained using the solute permeability coefficient [22,23]. Additionally, the effect of CP is estimated through correlations with the Sherwood and Péclet numbers. However, these parameters are related to the construction of membrane systems. Therefore, different values can be obtained in different membrane systems.

A new parameter, defined as the water transmission coefficient (η_WT_), was introduced [24]. The relationship between the water transmission coefficient, ECP, and ICP has been established and quantified, providing an effective and simplified way to evaluate the influence of CP on the water flux of FO membranes. The main purpose of this paper was to quantitatively evaluate the influence of ICP and ECP on the osmotic pressure differential using a new model and thus the water flux of the FO membrane under different operating conditions. Firstly, the experimental osmotic pressure and experimental water flux were measured under different types and concentrations of DSs, and the proportions of osmotic pressure drop arising from different kinds of DSs were quantified. Then, the influence of organic matter on FO membrane performance was investigated. Based on this, conditions including organic concentration, DS types, and membrane orientation were discussed to evaluate the effect on CP.

## 2. Materials and Methods

### 2.1. FO Membrane

The FO membrane used in this study was a fabric-reinforced TFC FO membrane provided by Dr. Zhao [25]. The as-prepared membrane consisted of a polyamide active layer and a fabric-embedded polysulfide support layer with an overall thickness of 60 μm. The tensile strength and Young’s modulus are about 90 MPa and 370 MPa, respectively. Furthermore, the membrane structural parameter is 370 μm. The FO membrane was kept in glycerol before the experiment to avoid irreversible shrinking upon drying.

### 2.2. Feed and Draw Solution

Humic acid (HA) was used as a model organic foulant to represent natural organic matter. The HA stock solution was prepared by dissolving 1 g of HA (Milwaukee, WI, USA) into 500 mL 0.01 mol/L NaOH solution. Then the solution was stored in a sterilized glass bottle (1000 mL) at 4 °C. The DS was sodium chloride solution (NaCl), calcium chloride solution (CaCl_2_), and magnesium chloride (MaCl_2_).

### 2.3. Experimental Setup and Method

**Figure 1:**
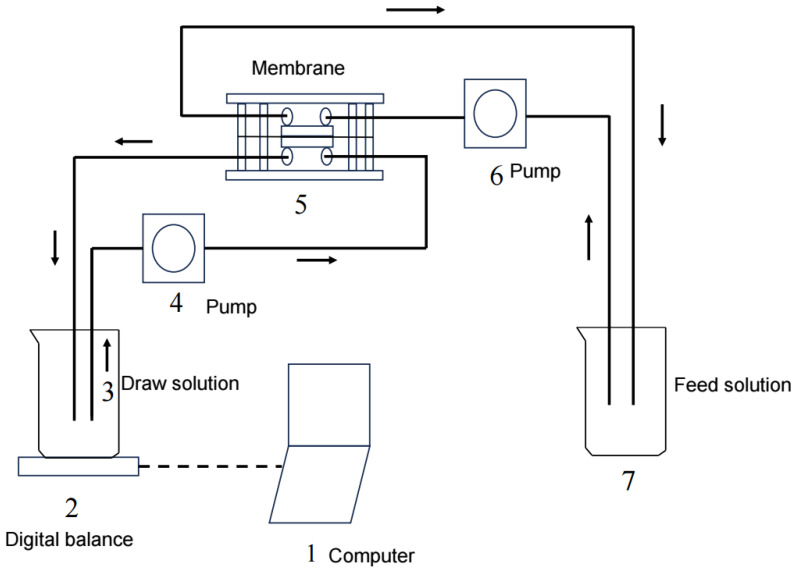
A schematic diagram of the FO system.

The experiments were performed using a custom-made flat organic glass cell with symmetric channels on both sides of the membrane, allowing for both the FS and DS to flow tangentially into the membrane. Each channel was 100 mm long, 45 mm wide, and 2 mm deep. The effective membrane area was about 0.004 m^2^. The FS and DS were separately circulated through the corresponding channels in a closed loop using two identical peristaltic pumps (YZ1515X, Baoding, China). Two temperature control devices (XMTD-204, Zhengzhou, China) were used to maintain a constant solution temperature of 25 ± 1 °C. The water flux was calculated using data recorded using a balance (BSA202S-CW, Gottingen, Germany). A schematic diagram of the FO system is shown in Figure 1. A virgin membrane was used in each experiment in order to compare the results under the same conditions. Prior to each experiment, the virgin membrane was soaked in deionized (DI) water for at least 24 h. The FO membrane was initially stabilized for 60–120 min with DI water as the FS and the corresponding solution as the DS. After that, experiments under different conditions were performed, and the water flux was determined. In the experiment, as water diffused through the membrane, the DS concentration slowly decreased. In order to maintain constant osmotic pressure, a high-concentration solution was continuously added to the DS to keep the concentration constant. All the tests were repeated three times.

In order to determine the real driving force, a static FO reactor was used in this study, as shown in previous research [24,26,27]. The static FO reactor was made of two transparent plexiglass chambers. In the middle of the two chambers lay a semi-permeable membrane with an effective area of 40 cm^2^. The upper chamber was filled with the FS, and the lower chamber was filled with the DS. There was a DS chamber outlet pipe linked to a manometer. Therefore, the water head difference between the two chambers was measured, and the osmotic pressure difference between the two chambers was calculated.

### 2.4. Analytical Method

#### 2.4.1. Experimental Water Flux

The mass change in the DS was continuously monitored using an electronic balance and a computer, and the experimental water flux was calculated using the following equation:

(1) $$J_{w,exp}=\frac{\Delta v}{\Delta t\times A_{m}}$$

where *J_w,exp_* is the experimental water flux, (L/m^2^·h); *Δv* is the mass change in the draw solution, g; *Δt* (h) is the interval time, h; *A_m_* is the membrane effective area, m^2^.

#### 2.4.2. Definition of $\eta_{\mathrm{MT}}$

The FO flux is usually given by Equation (2) [28]:

(2) $$J_{w}=A\times \sigma \left(\Delta \pi -\Delta P\right)$$

where *J_w_* is the FO water flux, L/m^2^·h (LMH); *A* is the membrane water permeability coefficient, m/s·Pa; *σ* is the osmotic reflection coefficient, dimensionless parameter; *∆P* is the applied hydraulic pressure, Pa; *∆π* is the osmotic pressure difference between the DS and FS.

CP combined with the FO process reduces the solute concentration difference across the membrane, thereby lowering its theoretical osmotic pressure to the effective osmotic pressure (*∆π_eff_*). Therefore, the flux can be calculated using Equation (3):

(3) $$J_{w,exp}=A\;\Delta \pi_{exp}=A\;\sigma \Delta \pi_{eff}=A\sigma \left(\pi_{DM}-\pi_{FM}\right)$$

Obviously, Equation (2) is valid only if the theoretical osmotic pressure is equal to the effective osmotic pressure, which means complete mixing in the two chambers. Equation (3) is difficult to apply across different FO systems. Thus, researchers have defined a new coefficient, *η_WT_* (the FO water transmission coefficient) [24], which is calculated using Equation (4):

(4) $$\eta_{WT}=\frac{J_{w,exp}}{J_{w,theoretical}}$$

When the value of $J_{w,exp}$ is equal to the value of $J_{w,theoretical}$, the value of $\eta_{\mathrm{MT}}$ is 1. Then,

(5) $$J_{w,theoretical}=A\;\Delta \pi_{theorecial}$$

further defined as

(6) $$J_{w,exp}=A\;\Delta \pi_{exp}=A\eta \Delta \pi_{theorecial}$$

and

(7) $$\eta_{WT}=\frac{\Delta \pi_{exp}}{\Delta \pi_{theorecial}}$$

Previous studies have shown that $\eta_{WT}$ can be a better parameter to describe the efficiency of the FO membrane system.

It is assumed that the solute can completely penetrate through the support layer, which means that the phenomenon of diluted external concentration polarization (ECP) would not occur on the outer surface of the support layer. Previous studies have proposed this hypothesis, which was considered applicable for membranes with a low water flux [29]. This assumption simplifies the analysis of the FO process and the value of CP. So, the osmotic pressure across the membrane can be expressed as follows:

(8) $$\Delta \pi_{th}=\pi_{D,b}-\pi_{F,b}\;$$

(9) $$\Delta \pi_{exp}=\sigma \Delta \pi_{eff}=\sigma (\pi_{D,m}-\pi_{F,m})\;$$

*Δπ_th_* is the theoretical osmotic pressure, and *Δπ_exp_* is the experimental osmotic pressure.

Dilutive ECP and concentrative ICP usually occur in pressure-retarded osmosis (PRO) mode, where the membrane active layer contacts the DS, which causes the DS concentration on the membrane surface to be lower than that of the bulk DS. Therefore, *Δπ_th_* is the summation of the osmotic pressure induced via ICP (*Δπ_exp_*) and the osmotic pressure induced via ECP. Therefore, in PRO mode,

(10) $$T_{Concentrative\;ICP}+\eta_{MT}+T_{Diluted\;ECP}+\frac{\eta_{WT}\left(1-\sigma \right)}{\sigma }=1$$

(11) $$\frac{\pi_{D,m}}{\pi_{D,b}}=e^{-\frac{J}{k}}=M_{1}<1$$

(12) $$\frac{\pi_{F,m}}{\pi_{F,b}}=e^{JK}=M_{2}>1$$

where *π_D,b_* is the osmotic pressure of the DS, *π_F,b_* is the osmotic pressure of the FS, *π_D,m_* is the osmotic pressure of the DS on the active layer surface, and *π_F,m_* is the osmotic pressure of the FS at the interface between the active layer and the support layer. *M_1_* is the coefficient of dilutive ECP, unitless. *M_2_* is the coefficient of concentrative ICP, unitless. *J* is the experimental water flux, m·s^−1^. *k* is the mass transfer coefficient, m·s^−1^. *K* is the resistance coefficient of solute diffusion in the support layer, which can be calculated as follows [30]:

(13) $$K=\frac{S}{D\;}=\frac{t\tau }{D\varepsilon }$$

where *S* is the membrane structure parameter, m; *D* is the effective diffusion coefficient of solute in the support layer, m^2^/s; and *t*, *τ*, and *ε* are the thickness (m), bending degree (unitless), and porosity (unitless) of the support layer. It is worth noting that this study used the effective diffusion coefficient of water in the support layer, as in previous studies [24,31].

Similarly, in FO mode, dilutive ICP occurs on the inner surface of the support layer, and concentrative ECP occurs on the active layer. Hence,

(14) $$T_{Dilute\;ICP}+\eta_{WT}+T_{Concentrative\;ECP}+\frac{\eta_{WT}\left(1-\sigma \right)}{\sigma }=1$$

(15) $$\frac{\pi_{D,m}}{\pi_{D,b}}=e^{-JK}=M_{3}<1$$

(16) $$\frac{\pi_{F,m}}{\pi_{F,b}}=e^{\frac{J}{k}}=M_{4}>1\;\;\;$$

where *π_D,b_* is the bulk osmotic pressure of the draw solution, *π_F,b_* is the bulk osmotic pressure of the feed solution, *π_D,m_* is the osmotic pressure of the draw solution at the interface between the active layer and the support layer, *π_F,m_* is the osmotic pressure at the side of the active layer. *M_3_* is the coefficient of diluted ICP, unitless; and *M_4_* is the coefficient of concentrative ECP, unitless.

The effect of ICP is related to water flux, in PRO mode,

(17) $$K=\frac{1}{J}ln\frac{B+A\sigma \pi_{D,m}-J}{B+A\sigma \pi_{F,b}}$$

and in FO mode,

(18) $$K=\frac{1}{J}ln\frac{B+A\sigma \pi_{D,b}}{B+A\sigma \pi_{F,m}+J}$$

where *B* is solute permeability, m/s; *A* is the water permeability coefficient, LMH/bar; and *σ* is the osmotic reflection coefficient, unitless. According to Equations (11), (12), (15)–(18),

(19) $$M_{2}=\frac{B+AM_{1}\sigma \pi_{D,b}-J}{B+A\sigma \pi_{F,b}}\;\left(\mathrm{PRO}\;\mathrm{mode}\right)$$

(20) $${\frac{1}{M}}_{3}=\frac{B+A\sigma \pi_{D,b}}{B+AM_{4}\sigma \pi_{F,b}+J}\;\left(\mathrm{FO}\;\mathrm{mode}\right)$$

According to Equations (7)–(9),

(21) $$\eta_{WT}=\frac{\sigma \left(C_{D,b}M_{1}-C_{F,b}M_{2}\right)}{C_{D,b}-C_{F,b}}\;\left(\mathrm{PRO}\;\mathrm{mode}\right)$$

(22) $$\eta_{WT}=\frac{\sigma \left(C_{D,b}M_{3}-C_{F,b}M_{4}\right)}{C_{D,b}-C_{F,b}}\;\left(\mathrm{FO}\;\mathrm{mode}\right)$$

When *σ* is 1, Equations (21) and (22) can be given as follows:

(23) $$\eta_{WT}=\frac{C_{D,b}M_{1}-C_{F,b}M_{2}}{C_{D,b}-C_{F,b}}\;\left(\mathrm{PRO}\;\mathrm{mode}\right)$$

(24) $$\eta_{WT}=\frac{C_{D,b}M_{3}-C_{F,b}M_{4}}{C_{D,b}-C_{F,b}}\;\left(\mathrm{FO}\;\mathrm{mode}\right)$$

The values of $\eta_{WT}$, $C_{D,b}$, $C_{F,b}$, $M_{1}$, $M_{3}$, $M_{2}$, and $M_{4}\;$ can be calculated using the above formulas. The model developed by Rong et al. can be used to evaluate the effects of ECP, ICP, and η_WT_ in the FO process [24].

Specifically, the effect of dilutive ECP (*T_DECP_*) and concentrative ICP (*T_CICP_*) in PRO mode on the theoretical osmotic pressure drop can be calculated via Equations (25) and (26):

(25) $$T_{Dilute\;ECP}=\frac{C_{D,b}-\;C_{D,b}\;M_{1}}{C_{D,b}-C_{F,b}}\times 100\%$$

(26) $$T_{Dilute\;ECP}=\frac{C_{D,b}-C_{D,b}\;M_{1}}{C_{D,b}-C_{F,b}}\times 100\%$$

The effect of dilutive ICP (*T_DICP_*) and concentrative ECP (*T_CECP_*) in FO mode on the theoretical osmotic pressure drop can be calculated via Equations (27) and (28):

(27) $$T_{Dilute\;ICP}=\frac{C_{D,b}-\;C_{D,b}\;M_{3}}{C_{D,b}-C_{F,b}}\times 100\%$$

(28) $$T_{Concentrative\;ECP}=100\%-T_{Dilute\;ICP}-\eta_{WT}$$

## 3. Results and Discussion

### 3.1. Relationship Between $\eta_{WT}$ and CP, and Their Implications

Table 1 shows the water flux of the FO membrane under different types of DSs. The water flux increased with the concentration of the DS, indicating that the increase in the osmotic pressure difference significantly improved the driving force and thus the water flux of the forward osmosis penetration process. The rate of increase in the water flux was much lower than the theoretical osmotic pressure difference. When the theoretical osmotic pressure difference increases twofold, the corresponding water flux increases only about 17–42%, suggesting that osmotic pressure loss across the membrane increases with the DS concentration. This inconsistency may be attributed to deviations in membrane semi-permeability, concentration polarization, reverse solute flux, and membrane resistance [23,32,33].

**Figure 2:**
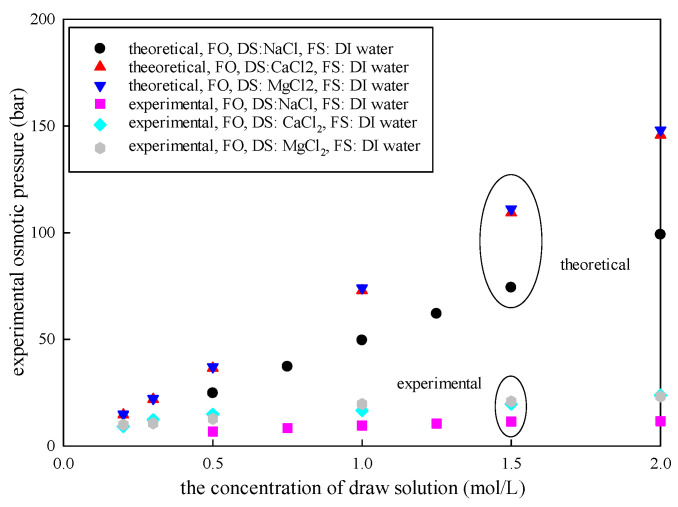
The variation between $\Delta \pi_{exp}$ and $\Delta \pi_{theorecial}$ with DS concentration (FO mode; draw DS was NaCl, CaCl_2,_ and MgCl_2_; concentration varied in the range of 0.2–2.5 mol/L; FS was DI water, 25 °C).

According to van’t Hoff’s equation, the theoretical osmotic pressure is directly proportional to the solution concentration in a dilute solution [21,34]. This indicates that higher DS concentration causes greater osmotic pressure. As shown in Table 1 and Figure 2, $\Delta \pi_{theorecial}$ increased from 24.5 bar to 99.1 bar as the concentration of NaCl increased from 0.5 mol/L to 2 mol/L. Meanwhile, $\Delta \pi_{exp}$ increased from 6.71 bar to 11.59 bar. However, there was a nonlinear relationship between the experimental osmotic pressure and the DS concentration. A similar effect was observed when the DS was CaCl_2_ or MgCl_2_. Notably, that there was also a significant difference between $\Delta \pi_{exp}$ and $\Delta \pi_{theorecial}$.

The difference between $\Delta \pi_{exp}$ and $\Delta \pi_{theorecial}$ becomes larger with the DS concentration. The results showed that the experimental osmotic pressure is only 14–68% of the theoretical osmotic pressure in the FO system.

**Figure 3:**
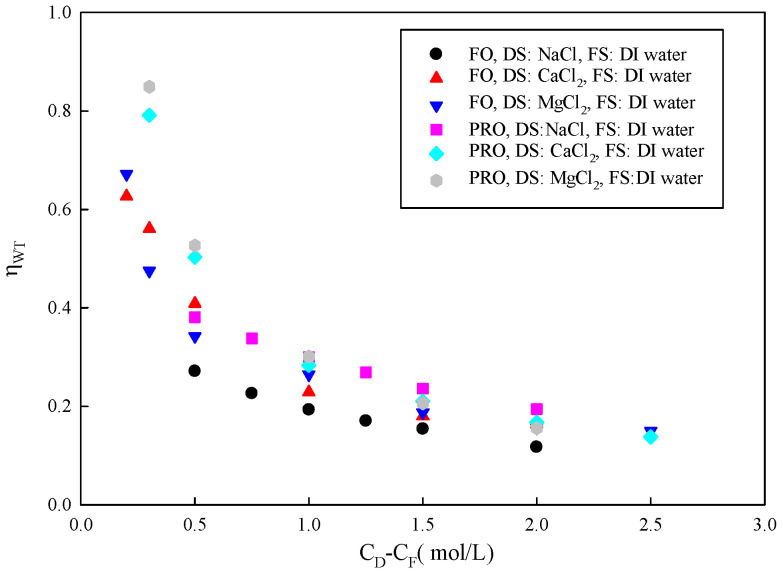
Relationship between $\eta_{\mathrm{WT}}$ and concentration difference across the membrane (C_D_-C_F_).

$\eta_{\mathrm{WT}}$ is the ratio of experimental water flux to the maximum possible water flux passing through the membrane in an imaginary membrane system that has no FO effects at all (the difference in the chemical potential between the DS and FS is zero, but with an applied ΔP equal to $\Delta \pi_{theorecial}$). Therefore, it successfully simulates substance transport in FO membranes. A high value of $\eta_{\mathrm{WT}}$ represents the FO membrane’s high mass transfer efficiency. Figure 3 shows the relationship between $\eta_{\mathrm{WT}}$ and concentration gradient. When the FS is DI water, the value of $\eta_{\mathrm{WT}}$ decreases with increasing DS concentration. Both $J_{theorecial}$ and $J_{exp}$ increase with an increased concentration gradient across the membrane. It seems that water transfer efficiency should also have risen. In fact, $\eta_{\mathrm{WT}}$ gradually decreases. The reason may be that the solute reverse flux increases with increasing DS concentration when using an electrolyte as the solute. Due to the opposite direction of motion, the solute may interfere with water transmission [35]. In addition, solutes may be adsorbed in the membrane, blocking water permeation, increasing membrane resistance, and reducing forward mass transfer efficiency.

**Figure 4:**
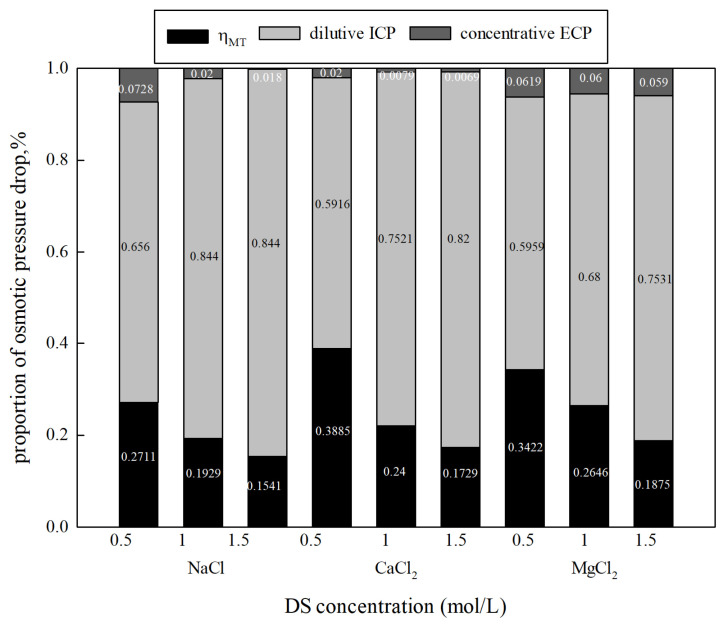
CP’s influence on osmotic pressure drop under various concentrations and types of DSs (FS: DI water, FO mode).

In order to understand the negative influence of CP, the proportions of osmotic pressure drop caused by different CPs can be calculated using a mathematical simulation model, such as Equations (26) and (27). From Figure 4, it can be seen that as the concentration of NaCl, CaCl_2_, and MgCl_2_ increased from 0.5 mol/L to 1.5 mol/L, the proportion of dilutive ICP showed a significant increase from 65.5% to 84.4%, 59.16% to 82.01%, and 59.59% to 75.31%, and $\eta_{\mathrm{WT}}$, a parameter representing water permeation efficiency, decreased from 27.11% to 15.41%, 38.85% to 17.29%, and 34.22% to 18.75%, respectively. There are differences in both the theoretical and experimental osmotic pressure caused by different types of draw solutions at the same concentration. Therefore, η_WT_ and CP in different types of draw solutions are differentiated. The results also suggest that the concentration gradient in the boundary layers increases as the DS concentration increases, resulting in extensive polarization of dilutive ICP. Concentrative ECP has little effect on the osmotic pressure drop, accounting for less than 8% in all tests. ICP plays a more prominent role in the osmotic pressure drop at higher DS concentrations. However, the influence of ECP cannot be ignored, as shown in previous studies, especially in high-salinity solutions [21]. Therefore, both ECP and ICP could be responsible for the reduction in osmotic pressure.

### 3.2. Influence of Organic Matter on $\eta_{WT}$ and CP

#### 3.2.1. Influence of Organic Concentration

**Figure 5:**
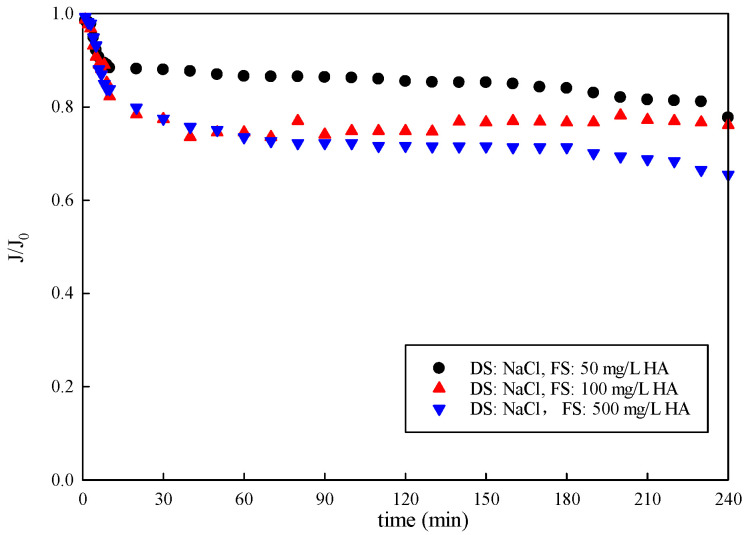
Variation in membrane-normalized water flux with time under FO mode.

The results of the normalized water flux with an organic concentration in the feed solution are displayed in Figure 5. The water flux decreased with the increasing organic concentration in the FS in FO mode. The water flux gradually decreased to 77.70%, 76.13% and 65.53% at organic concentrations of 50 mg/L, 100 mg/L, and 500 mg/L, respectively. Flux decline dropped sharply with the organic concentration of 500 mg/L compared to 50 mg/L and 100 mg/L. As the organic concentration in the FS increased, the water flux decreased. Furthermore, the trend of water flux decline was similar, with a rapid decrease in the beginning (initial 30 min) followed by a slower phase with constant water flux loss. It reveals different fouling mechanisms governing the two filtration stages. Foulant–membrane interactions determined fouling behavior in the beginning, and foulant–foulant interactions dictated the performance in the later slow phase. Organics may penetrate the membrane, causing pore plugging, or absorb on the membrane surface with water permeation, hindering water transmission. A cake layer was formed and accumulated on the membrane surface, leading to a further decrease in the flux [36,37,38].

**Figure 6:**
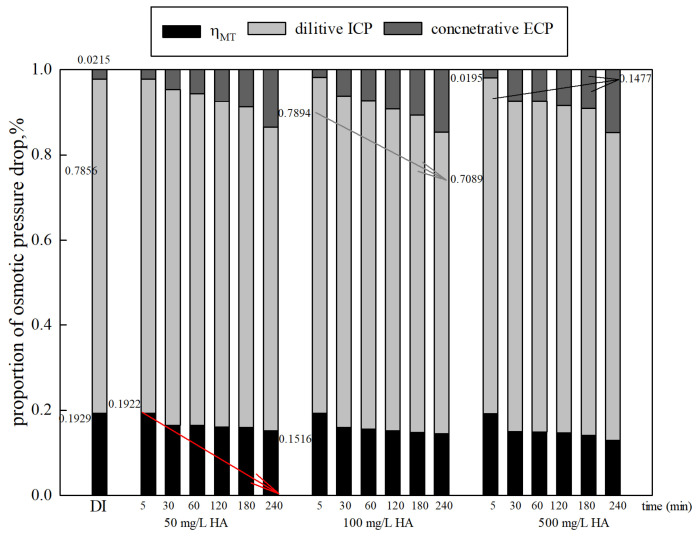
Proportions of osmotic pressure drop with various organic concentrations (DS 1.0 mol/L NaCl, FO mode).

The variation in $\eta_{\mathrm{WT}}\;$ shows a similar trend to the water flux. The solute in the FS increases, causing a decline in the water transfer efficiency of the FO membrane. Both the proportion of dilutive ICP and $\eta_{\mathrm{WT}}\;$ decrease, and the proportion of concentrative ECP increases over time, as shown in Figure 6. The reduction in ${\;\eta }_{\mathrm{WT}}$ was only about 4.06–6.25% during operation. The proportion of dilutive ICP increased by 0.29% as the organic concentration increased from 50 mg/L to 500 mg/L. The change in the proportion of concentrative ECP cannot be ignored. The increase was about 12%. Furthermore, it is worth noting that CP increased along with the FS concentration. The results can be explained as follows: when a certain concentration of FS is used instead of DI feed water, concentrative ECP emerged on the membrane active layer, while the high DS concentration also led to more severe dilutive ICP on the membrane support layer [39,40]. Furthermore, fouling and gel or cake layers caused by organics also have a negative impact on membrane and mass-transfer characteristics [41]. They may increase membrane thickness and roughness, reduce membrane porosity, and cause higher filtration resistance, reducing the effective osmotic pressure difference and subsequently hindering the membrane water flux.

#### 3.2.2. Influence of DS Types

**Figure 7:**
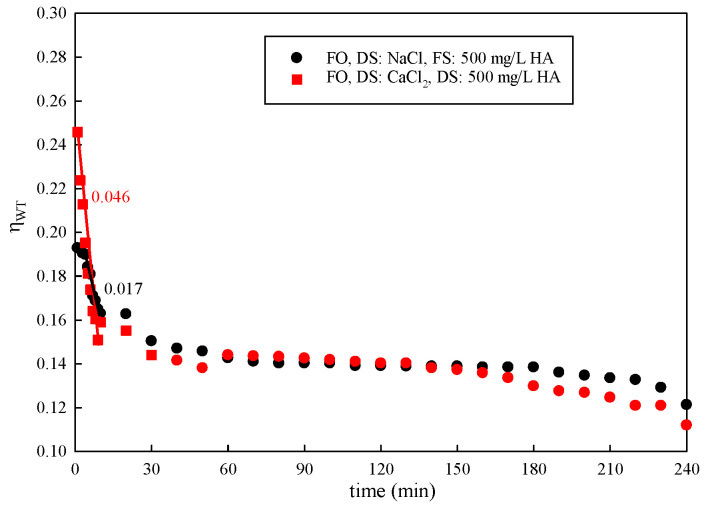
Variation in $\eta_{\mathrm{WT}}$ with time under different types of DSs.

Experiments were conducted with different types of DSs. The results are shown in Figure 7. The membrane water transfer efficiency was improved via the high osmotic pressure provided by CaCl_2_. However, the water transfer efficiency significantly decreased with the use of CaCl_2_ as the DS. In the initial 30 min, the water transfer efficiency decreased sharply. The decline rate of $\eta_{\mathrm{MT}}$ using CaCl_2_ (0.046) as the DS was almost three times higher than that of NaCl (0.017). After this, it decreased slowly. Upon completion of the 240 min study, $\eta_{\mathrm{WT}}$ gradually decreased to 66.94% and 44.02% with a DS concentration of 1 mol/L NaCl and CaCl_2_, respectively. The solute’s reverse permeation behavior is common during the FO process. Ca^2+^ binds with carboxylic acid groups in natural organic matter, leading to a significant drop in water transfer efficiency [42,43].

**Figure 8:**
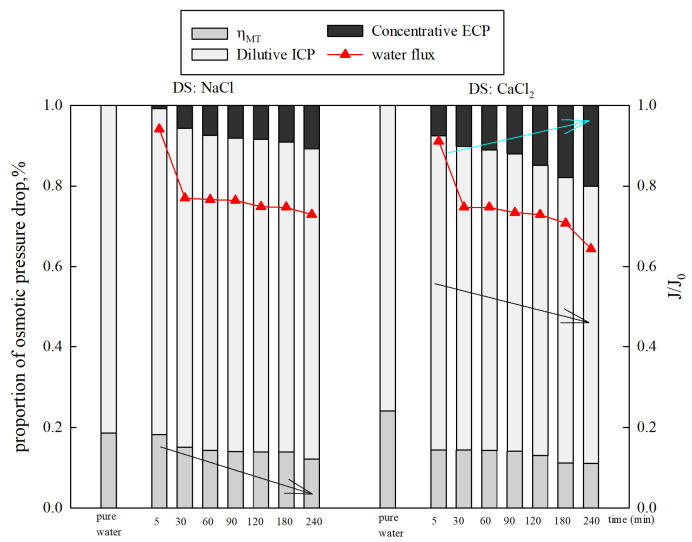
The variation in concentration polarization under different types of DSs.

Figure 8 displays the proportions of osmotic pressure drop caused by CP under different types of DSs. It was found that the dilutive ICP was responsible for 81.44% (NaCl) and 80.09% (CaCl_2_) of the osmotic pressure loss in the initial stage. Then, the proportions of osmotic pressure drop caused by dilutive ICP slowly decreased from 81.44% to 77.09% (NaCl) and 80.08% to 68.98% (CaCl_2_). The influence of concentrative ECP on the osmotic pressure drop increased from 0.69% to 10.77% (NaCl) and 7.46% to 19.98% (CaCl_2_), while $\eta_{\mathrm{WT}}$ gradually decreased from 19.29% to 12.13% (NaCl) and 24.01% to 11.04% (CaCl_2_). The results show that dilutive ICP is more important than the concentrative ECP on the osmotic pressure drop under FO mode.

#### 3.2.3. Effect of Membrane Orientation

**Figure 9:**
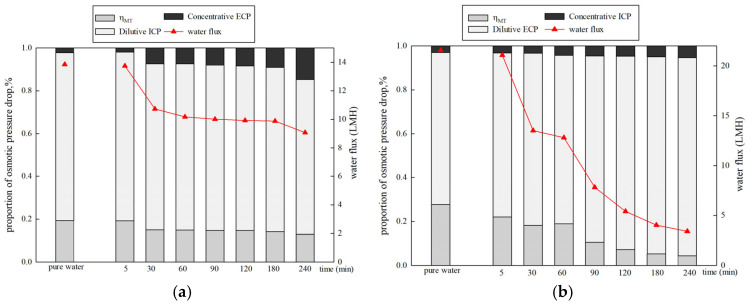
Proportions of osmotic pressure drop and water flux over time under different membrane orientations. (**a**) DS 1 mol/L NaCl, FS: 500 mg/L HA, FO mode (**b**) DS 1 mol/L NaCl, FS: 500 mg/L HA, PRO mode.

The membrane performance when using 1 mol/L NaCl as the DS and 500 mg/L HA as the FS is shown in Figure 9. The pure water flux in PRO mode (21.55 LMH) is higher than that of FO mode (13.84 LMH). This is due to less severe concentrative ICP (0.036) in PRO mode compared to dilutive ICP (0.78) in FO mode [39]. In the presence of organics, a greater decrease in the water flux is observed in PRO mode compared to FO mode. In FO mode, the water flux decreased to 65.53% at the end of the filtration. However, in PRO mode, the water flux declined to 15.74%. Figure 9 also displays the proportions of osmotic pressure drop caused by CP in different membrane orientations. Dilutive ICP and concentrative ECP occur in FO mode, while dilutive ECP and concentrative ICP occur in PRO mode [24]. Dilutive ICP shows a significant negative effect on osmotic pressure in FO mode. The proportion of dilutive ICP decreased by 6.22%, while the proportion of concentrative ECP increased by 12.62%. At the same time, $\eta_{\mathrm{MT}}$ declined by 6.4%. However, in PRO mode, both the proportions of dilutive ECP and concentrative ICP increased from 69.26% to 90.23% and 3.04% to 5.35%. Only $\eta_{\mathrm{WT}}$ gradually decreased by 23.29%. The results reveal that dilutive ECP is dominant in PRO mode. The proportions of the osmotic pressure drop caused by CP in PRO mode are higher than those in FO mode. The large pore sizes, high porosity, and rough structure of the support layer enable significant pore blocking and the formation of a cake layer in the support layer in PRO mode, enhancing CPs and causing a sharp decline in the flux (water flux decreases by 84.26%). Previous literature has reported that dilutive ECP in the boundary layer and ICP can significantly undermine the performance of a system [20,21].

## 4. Conclusions

In this study, the water transmission coefficient (η_WT_) was introduced, and the relationship between η_WT_, ECP, and ICP was established and quantified. Furthermore, the effects of both ICP and ECP on the water flux and osmotic pressure drop under different concentrations were quantitatively investigated. The influence of organic matter concentration, DS types, and membrane orientation was also discussed. The following conclusions were drawn:

(a) η_WT_ decreased with the increase in C_D_-C_F_ in the FO system under the influence of different DS types and membrane orientations. Both ECP and ICP were capable of reducing osmotic pressure. However, ICP was dominant when DI water was used as the FS in FO mode.
(b) With the increased concentration of organic compounds in the FS, the reduction in water flux and the adverse effect of CP increased in FO mode. CaCl_2_, when used as the DS, resulted in a higher osmotic pressure and a higher η_WT_ compared to NaCl. A high reduction in water transfer efficiency over filtration time was also obtained. A higher pure water flux and water flux decline were observed in PRO mode than in FO mode. In FO mode, both the proportion of dilutive ICP and η_WT_ decreased, while the proportion of concentrative ECP increased over time. However, in PRO mode, the proportions of dilutive ECP and concentrative ICP increased, while η_WT_ decreased. Dilutive ICP was observed to have a significant negative effect on osmotic pressure in FO mode, while dilutive ECP was dominant in PRO mode.
(c) The FO water transmission coefficient, η_WT_, defined as J_w, exp_/J_w, theoretical_, provided a simpler way to understand and compare the efficiency of FO systems. In particular, it has more practical value in predicting FO water flux and performance under different conditions. In addition, model optimization to enhance its reliability and applicability will be studied in the future.

## Figures and Tables

**Table 1:** Theoretical osmotic pressure and experimental water flux under different types of DSs (FS was DI water).

Type of DS	DS Concentration (mol/L)	∆*π*_the_ (bar)	*J_exp_* (LMH)
NaCl	0.5	24.78	9.75
	0.75	37.16	12.18
	1	49.55	13.84
	1.25	61.94	15.25
	1.5	74.33	16.61
	2	99.1	16.83
CaCl_2_	0.2	14.57	9.4
	0.3	21.86	12.57
	0.5	36.44	15.24
	1	72.88	17.53
	1.5	109.32	20.24
	2	145.75	24.48
MgCl_2_	0.2	14.81	8.88
	0.3	22.22	9.29
	0.5	37.03	11.93
	1	74.07	17.88
	1.5	111.10	18.78
	2	148.13	20.98
	2.5	185.17	24.95

## Data Availability

The original contributions presented in this study are included in the article. Further inquiries can be directed to the corresponding authors.
